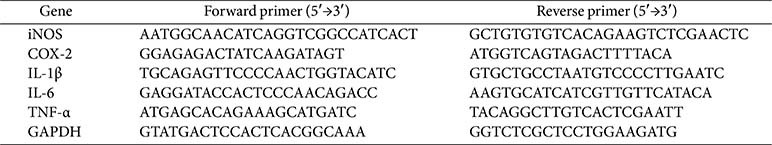# Cannabigerol Exerts *In Vivo* and *In Vitro* Anti-Inflammatory Effects via Inhibition of the MAPK and NF-κB Pathways

**DOI:** 10.4014/jmb.2509.09034

**Published:** 2025-12-18

**Authors:** Jong-Hui Kim, Min Hong, Joon-Hee Han, Byeong Ryeol Ryu, Jung Dae Lim, Keun-Cheol Kim, Chang-Hyeug Kim, Soo-Ung Lee, Tae-Hyung Kwon

**Affiliations:** 1 Institute of Biological Resources, Chuncheon Bioindustry Foundation, Chuncheon 24232, Republic of Korea; 2 Institute of Cannabis Research, Colorado State University-Pueblo, 2200 Bonforte Blvd., Pueblo, CO, 81001-4901, USA; 3 Department of Bio-Health Convergence, Kangwon National University, Chuncheon, 24341, Republic of Korea; 4 Department of Bio-Functional Material, Kangwon National University, Samcheok 25949, Republic of Korea; 5 Department of Biological Sciences, Kangwon National University, Chuncheon 24341, Republic of Korea

**Keywords:** Cannabigerol, cannabis sativa, pink pepper, anti-inflammatory, paw edema

## Abstract

*Cannabis sativa* L. has a long history of use and contains more than 80 cannabinoids. However, although cannabigerol (CBG), which acts as a biosynthetic precursor of its most abundant phytocannabinoids, has anti-inflammatory effects, the exact mechanism of action remains underexplored. In this study, we explored the anti-inflammatory potential of CBG to assess its potential for therapeutic and industrial applications. CBG was extracted from the cannabis cultivar ‘Pink Pepper’ *In vitro* assays were performed via RAW 264.7 mouse macrophages stimulated with lipopolysaccharide, and *in vivo* efficacy was evaluated through a carrageenan-induced paw edema mouse model to confirm the activity of CBG in acute inflammation. Nitric oxide production, mRNA, and protein expression of inflammatory mediators were suppressed by CBG treatment in a process downregulated through the MAPK and NF-κB pathways. Although paw edema was not statistically significantly reduced, oral administration of CBG suppressed the expression of COX-2, iNOS, TNF-α, IL-1β, and IL-6 in the carrageenan-induced mouse model. CBG has been demonstrated to exert significant anti-inflammatory effects via modulation of key inflammatory mediators and signaling pathways in both *in vivo* and *in vitro* models. Our findings further support the potential of CBG as a bioactive compound for further anti-inflammatory research.

## Introduction

*Cannabis sativa* L, commonly known as hemp, has been used for medicinal purposes for centuries [1]. Since the identification of the molecular structure of its primary psychoactive compound, Δ^9^-tetrahydrocannabinol (Δ^9^-THC), nearly 50 years ago, cannabinoids have been the subject of extensive chemical and biological investigations [2]. Cannabinoids constitute a group of compounds that modulate the endogenous cannabinoid system by binding primarily to the cannabinoid receptors CB_1_ and CB_2_ [3]. In addition to these interactions, cannabinoids influence a variety of other receptors [4]. So far, more than 80 distinct cannabinoids have been reported, with increasing attention directed toward their pharmacological properties and therapeutic potential [5]. Cannabinoids have demonstrated promising applications in the treatment of various conditions, such as glaucoma, depression, neurological disorders, Alzheimer’s disease, HIV/AIDS, and cancer-related symptoms [2].

**Fig. 1:**
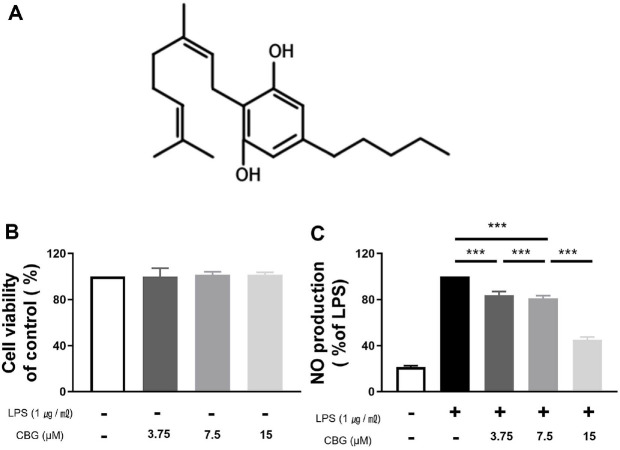
Structure of CBG and effect of CBG on LPS-stimulated RAW 264.7 cells. (**A**) Chemical structure of CBG. (**B**) Viability of RAW 264.7 cells treated with various concentrations of CBG compared with that of the control. (**C**) Results of inhibition of nitric oxide production rate in groups treated with various concentrations of CBG compared with that in the LPSonly treatment group. Data represent the means ± SD in triplicate. The *p*-value was calculated based on LPS-only data using ANOVA and Tukey’s post-hoc test (***p < 0.001). LPS: lipopolysaccharide, CBG: cannabigerol.

Cannabigerol (CBG) (Fig. 1A) functions as a biosynthetic precursor to the most abundant phytocannabinoids [6]. The cannabinoid biosynthetic pathway begins with olivetolic acid and geranyl pyrophosphate, which combine to form cannabigerolic acid (CBGA) [7]. CBGA subsequently serves as the central precursor for other major cannabinoids, including Δ^9^-tetrahydrocannabinolic acid (Δ^9^-THCA), cannabidiolic acid (CBDA), and cannabichromenic acid [7]. CBG is produced from CBGA via nonenzymatic decarboxylation. Although these cannabinoids share a common precursor, the physiological effects of each cannabinoid vary considerably [6].

CBG activates α_2_-adrenergic receptors, inhibits serotonin 1A (5-HT_1_A) and CB_1_ receptors, and binds to cannabinoid receptor 2 (CB_2_ receptors), potentially conferring neuroprotective effects [6]. Moreover, such neuroprotective properties of CBG have been reported in studies employing both cellular and animal models of neurological conditions, including Parkinson’s disease and multiple sclerosis [7, 8]. CBG has also demonstrated therapeutic efficacy in gastrointestinal disorders, including colon cancer and colitis, as evidenced in mouse models [9]. In addition, CBG exhibits pharmacological activity in anticancer [10, 11], antibacterial [12], and metabolic diseases [13]. Its anti-inflammatory effects have been attributed to its ability to suppress colitis-induced reactive oxygen species in the intestinal epithelial cells and to regulate redox homeostasis by inhibiting cytokine production expression [7, 14, 15]. However, the exact mechanism has not been identified and warrants further research.

Inflammation is a critical physiological response aimed at limiting tissue damage and promoting healing [16]. This complex process pertains to the expression of diverse proteins and cytokines, regulated immune cell recruitment, and the activation of multiple signaling pathways [17]. However, uncontrolled or excessive inflammatory responses may cause collateral injury to tissues, drive the onset or progression of various pathological conditions, and interfere with neurotransmitter signaling in the brain, thereby altering motivation-related behaviors and inducing anhedonia [18]. Therefore, compounds capable of downregulating inflammatory markers hold promise as potential anti-inflammatory agents.

Macrophages are major inflammatory cells that play a central role in inflammatory responses. Lipopolysaccharide (LPS), a component of the outer membrane of gram-negative bacteria, activates Toll-like receptor 4 (TLR4) of macrophages, thereby initiating immune signaling [19]. Activated macrophages produce excessive amounts of inflammatory mediators, including nitric oxide (NO), prostaglandins (PGs), and proinflammatory cytokines, such as tumor necrosis factor-α (TNF-α), interleukin-1β (IL-1β), and interleukin-6 (IL-6), primarily via the nuclear factor-kappa B (NF-κB) and mitogen-activated protein kinase (MAPK) signaling pathways [20].

We explored the anti-inflammatory potential of CBG extracted from the novel Korean hemp cultivar ‘Pink Pepper’ using both *in vitro* and *in vivo* models. This specific cultivar was bred in Korea for medicinal application, and displays elevated CBDA amounts and comparatively low amounts of Δ^9^-THCA. It also flowers independent of photoperiod, and shows robust adaptability to both indoor and outdoor environments. The whole-genome assembly of the Pink Pepper cultivar is available in GenBank (GCA_029168945.1) [21]. Comprehensive annotations, including gene structure and predicted genomic functions, are available in the Figshare database [22].

In the present work, we conducted *in vitro* assays employing RAW 264.7 cells and a murine macrophage, while *in vivo* response was assessed through a murine paw edema model induced by carrageenan to determine CBG’s potential against acute inflammation. In addition, the molecular and genetic mechanisms underlying CBG’s anti-inflammatory effects were elucidated, with an aim to demonstrate the pharmacological advantages of CBG as an anti-inflammatory agent and assess its potential for therapeutic and industrial applications.

## Materials and Methods

### Reagent

RAW 264.7 cells were purchased from the American Type Culture Collection (TIB-71; ATCC, USA). LPS (L6011) and λ-carrageenan (9064-57-7) were purchased from Sigma-Aldrich (USA), and DMEM cell culture media, fetal bovine serum (FBS), penicillin, and streptomycin were purchased from Gibco BRL (Invitrogen Co., USA). A Cell Counting Kit-8 (CCK-8) was purchased from Dojindo Molecular Technologies (USA). The NO detection kit and PRO-PREP were purchased from iNtRON (Republic of Korea). RNA was isolated with a commercial RNA isolation kit (12183018A; Thermo Fisher Scientific,). The utilized reverse transcription master mix was the High-Capacity RNA-to-cDNA Kit (Applied Biosystems, Thermo Fisher). RT- PCR was performed using FG SYBR Green PCR Master Mix (Applied Biosystems) All primary antibodies, iNOS, COX-2, TNF-α, IL-6, IL-1β, Erk1/2, p-Erk1/2, JNK, p-JNK, p38, p-p38, NF-κB, p-NF-κB, and β-actin, were purchased from Cell Signaling (USA). *In vivo* protein expression was measured using an ELISA kit, and the products are as follows: iNOS (MBS261100, MyBioSource, USA), COX-2 (MBS269104, MyBioSource), TNF-α (BMS607-3, Invitrogen, USA), IL-1β (SMLB00C-1, R&D Systems, USA), and IL-6 (ab100713, Abcam, UK).

### Plant Materials

In this study, Pink Pepper, a new cultivar of *C. sativa* L., was used. This cultivar was developed by Lim [21] and cultivated in an outdoor experimental field (latitude 37°53′03.300′′N, longitude 127°44′03.800′′E) of the Chuncheon Bio-industry Foundation in Chuncheon, Gangwon Province, Republic of Korea. Harvested hemp was dried at 45°C for 50 h using a hot air dryer. The dried hemp was subsequently ground using an 80-mesh grinder and stored at 23°C and 14% relative humidity.

### CBG Extraction

Dried and pulverized hemp (1,000 g) was injected into a 10 L supercritical fluid extraction (SFE) reactor. The SFE was conducted for 120 min under the following conditions: carbon CO_2_ flow rate of 300.0 g/min, sample chamber temperature of 60°C, separator temperature of 30°C, and system pressure of 470 bar (Phos-enthech, SFE-10L, Republic of Korea). Subsequently, 99% ethanol was added to the extract at a ratio of 1:10, mixed, and then frozen at −72°C for 20 h.

### CBG Purification

The filtered extract was concentrated for 60 min at 70 bar and 45°C via a rotary evaporator (R-220SE, Büchi, Switzerland) to remove residual ethanol. The concentrated sample was purified for the first time using centrifugal partition chromatography. A solvent system of n-hexane : ethyl acetate : methanol : water (1:1:1:1, v/v/v/v) was used for the purification. The flow rate was 10 ml/min, and the rotation speed was maintained at 400 ×*g*. Each fraction was confirmed through HPLC analysis, and the fraction containing CBG was collected for subsequent purification. Secondary purification was performed using a Medium-Pressure Liquid Chromatography (MPLC) system (Biotage, SIO-1EV, USA). The solvent was removed by employing a vacuum concentrator to obtain high-purity CBG (>99%).

### CBG Crystallization

Purified CBG (≥99% purity) was crystallized to obtain a stable solid form. For crystallization, 1 ml of pentane was added per 2 g of CBG, and the mixture was completely dissolved at 30°C. The solution was kept at −4°C for 60 min to initiate crystallization and continuously stirred with a magnetic stirrer to sustain crystal formation. The formed crystals were washed with pre-cooled pentane (−4°C). During the washing step, the crystals were collected using a vacuum filtration apparatus while simultaneously removing residual solvent. The washed crystals were then dried at 25°C for 30 min to obtain highly pure crystalline CBG.

### Cell Culture and Viability Assay

RAW 264.7 cells were maintained in Dulbecco's modified Eagle's medium supplemented with 10% fetal bovine serum, and 1% antibiotic-antimycotic (15240 Gibco, Thermo Fisher). Cells were cultured at 37°C in 5% CO_2_ (Heracell 150i CO_2_ Incubator, Thermo Fisher). The proliferation and viability of the RAW 264.7 cells were confirmed using the Cell Counting Kit-8 assay. RAW264.7 cells were seeded at 1 × 10^4^ cells/well in 96-well plates and cultured for 18 h. The cells were then treated with CBG at concentrations of 3.75, 7.5, and 15 μM. Cell viability assay was conducted following the manufacturer’s protocol.

### Nitric Oxide Assay

To evaluate the anti-inflammatory activity of CBG, RAW 264.7 murine macrophage cells were seeded at a density of 2 × 10^5^ cells/well in 24-well culture plates. The cells were then stimulated with LPS at a concentration of 1 μg/ml and treated with CBG at concentrations of 3.75, 7.5, and 15 μM, and incubated for an additional 18 h. Thereafter, NO content was evaluated using a NO detection kit (iNtRON Biotechnology), and conducted following the manufacturer’s protocol.

### Reverse Transcription-Quantitative PCR (RT-qPCR)

Total RNA was reverse transcribed into complementary DNA (cDNA). Relative expression levels were analyzed by qPCR using oligonucleotide primers (Table 1) and measured with a LightCycler 480 (Roche, Germany).

### Preparation of Cell Lysates and Nuclear Fractions and Western Blot Analysis

Total protein was lysed using PRO-PREP in RAW 264.7 cells after CBG treatment. The protein concentration (μg/μl) was determined with the Pierce BCA Protein Assay (Thermo Fisher Scientific, USA). Isolated proteins were resolved by SDS-PAGE and validated through antibody–antigen interaction. Signal intensity proportional to protein concentration was subsequently assessed with a digital imaging system (LAS-4000, Fujifilm, Japan).

### Mouse Model of Paw Edema Triggered by λ-Carrageenan

C57BL/6 male mice aged 6–7 weeks were purchased from Central Lab Animal Inc. (Republic of Korea) and acclimated for one week prior to the experiment. Mice were randomly allocated into the test and control groups, with each experimental group consisting of nine animals. The mice were maintained and fed ad libitum on standard feed, with environmental conditions set at 23 ± 2°C, 55 ± 5% humidity, and a 12-h light/dark cycle. Oral CBG was administered at 1 and 10 mg/kg body weight. Acute paw inflammation was induced by subcutaneous injection of 0.5% λ-carrageenan (50 μl) into the right hind paw of the experimental group, whereas the control group received sterile saline (50 μl).

### Assessment of Inflammatory Factors in Mouse Paw Tissue

To extract proteins from tissues of 0.5% λ-carrageenan-induced paw edema models, lysed in PRO-PREP and was centrifuged at 15,000 ×*g* for 15 min at 4°C to separate the supernatant. Proteins in the supernatant were identified using ELISA according to the manufacturer's protocol.

### Data Analysis

All experiments were conducted independently and repeated at least three times. Data were expressed as mean± SD. The experimental results were visualized using GraphPad Prism (GraphPad 8.0.1). Statistical analyses were performed using one-way analysis of variance (ANOVA) to evaluate differences among multiple experimental groups, followed by Tukey’s post-hoc test for pairwise comparisons, validated at significance levels of 5%, 1%, and 0.1% (*p < 0.05, **p < 0.01, ***p < 0.001).

## Results

### CBG Did Not Affect the Viability of RAW 264.7 Cells

The cytotoxic effect of CBG was assessed through the CCK-8 assay. RAW 264.7 cells were treated with CBG at concentrations of 3.75, 7.5, and 15 μM, and cell viability was measured following incubation. Relative to the untreated control group, CBG did not affect the viability of RAW 264.7 cells. Even at the highest dose of 15 μM, no cytotoxicity was detected (Fig. 1B).

### CBG Inhibited the Overproduction of NO

RAW 264.7 macrophages were employed to evaluate the effect of CBG on LPS-induced, excessive NO production, which was considerably elevated in the LPS-treated group relative to LPS-untreated group (21.45 ± 1.10%). However, NO production was reduced to 83.69 ± 2.93%, 81.12 ± 1.88%, and 45.00 ± 2.20% at 3.75, 7.5, and 15 μM of CBG, respectively (Fig. 1C). Therefore, we confirmed that CBG inhibited LPS-induced NO overproduction in a concentration-dependent manner (Fig. 1C).

### CBG Downregulates the Transcription of Inflammation-Related Genes

**Fig. 2:**
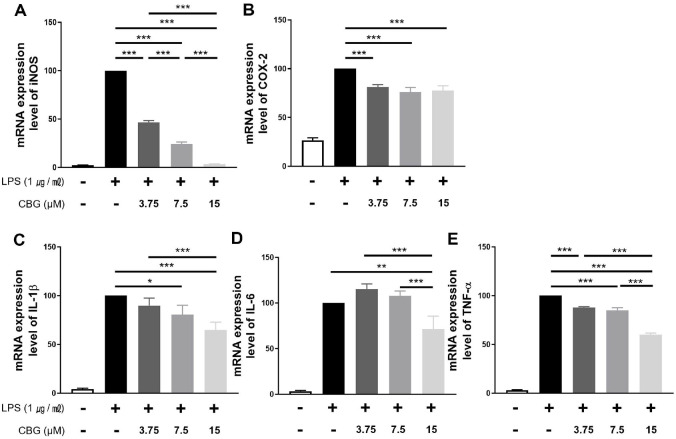
Inhibitory effect of CBG on mRNA expression of inflammatory factors. All samples except control were treated with 1 μg/ml of LPS. (**A**) iNOS, (**B**) COX-2, (**C**) IL-1β, (**D**) IL-6, and (**E**) TNF-α mRNA expression levels in groups treated with various concentrations of CBG compared with those in the LPS-only treatment group. Data represent the means ± SD in triplicate. The *p*-values were calculated based on LPS-only data using ANOVA and Tukey’s post-hoc test (*p < 0.05, *p < 0.01, ***p < 0.001.

The impact of CBG on the mRNA expression of inflammatory factor and cytokines was assessed in RAW 264.7 cells. CBG inhibited iNOS mRNA expression by 46.78 ± 1.55%, 24.30 ± 1.80%, and 3.37 ± 0.23% at concentrations of 3.75, 7.5, and 15 μM, respectively (Fig. 2A), indicating a concentration-dependent reduction. COX-2 mRNA levels showed inhibition of 81.11 ± 2.31%, 76.01 ± 4.05%, and 77.60 ± 4.22% at the same concentrations (Fig. 2B). However, this effect was not concentration-dependent (Fig. 2B). For IL-1β, CBG treatment at 3.75 μM revealed no significant inhibitory effect (89.84 ± 6.27%), whereas mRNA expression was reduced to 80.43 ± 8.01% and 64.68 ± 6.62% at 7.5 and 15 μM, respectively (Fig. 2C). IL-6 expression was significantly inhibited only at 15 μM (71.08 ± 11.75%) (Fig. 2D). TNF-α mRNA levels declined in a dose-responsive manner, showing reductions of 87.84 ± 0.69%, 85.04 ± 2.14%, and 59.94 ± 1.30% at 3.75, 7.5, and 15 μM, respectively (Fig. 2E). Overall, CBG downregulates the transcription of key inflammatory mediators with concentration-dependent effects.

### CBG Inhibits iNOS and Inflammatory Cytokines at the Protein Level

**Fig. 3:**
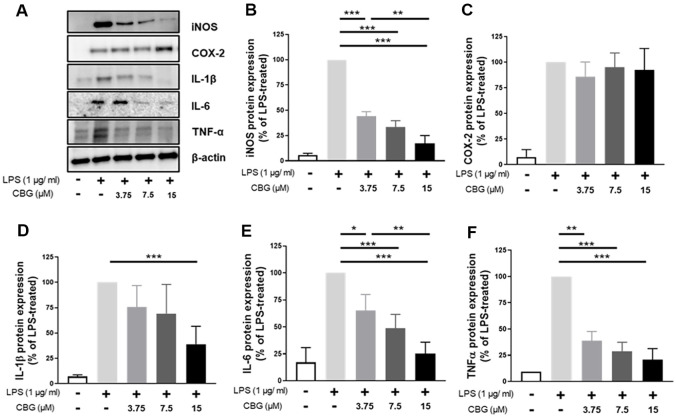
Inhibitory effect of CBG on protein expression of inflammatory factors. All samples except the control were treated with 1 μg/ml of LPS. (**A**) Western blot detection bands of each inflammatory factor protein in groups treated with various concentrations of CBG compared with those of (**B**) iNOS, (**C**) COX-2, (**D**) IL-1β, (**E**) IL-6, and (**F**) TNF-α protein expression levels in the LPS-only treatment group. All results were analyzed by normalizing to β-actin. The *p*-values were calculated based on LPS-only data using ANOVA and Tukey’s post-hoc test (*p < 0.05, **p < 0.01, ***p < 0.001).

The impact of CBG protein expression of inflammatory factors was evaluated via western blotting (Fig. 3A). Specifically, iNOS expression was inhibited by 89.45 ± 4.17%, 68.06 ± 4.49%, and 34.47 ± 2.76% at 3.75, 7.5, and 15 μM CBG, respectively (Fig. 3B). In contrast, COX-2 protein expression did not show a significant change in the 15 μM CBG-treated group compared to the LPS-only group (Fig. 3C). For IL-1β, no significant differences were observed at 3.75 and 7.5 μM CBG; however, a significant decrease of 38.92 ± 15.36% was observed at 15 μM CBG (Fig. 3D). In the case of IL-6, protein expression was inhibited in a concentration-dependent manner: 63.36 ± 12.73%, 48.61 ± 11.27%, and 25.22 ± 9.25% at 3.75, 7.5, and 15 μM CBG, respectively (Fig. 3E). TNF-α expression was also inhibited in a concentration-dependent manner, with reductions of 39.01 ± 6.10%, 28.78 ± 6.08%, and 20.86 ± 7.40% observed at 3.75, 7.5, and 15 μM CBG, respectively (Fig. 3F). In particular, TNF-α expression at 7.5 μM CBG was comparable to the level observed in the LPS-unstimulated group.

### CBG Regulates Inflammation by Inhibiting MAPK Pathways

**Fig. 4:**
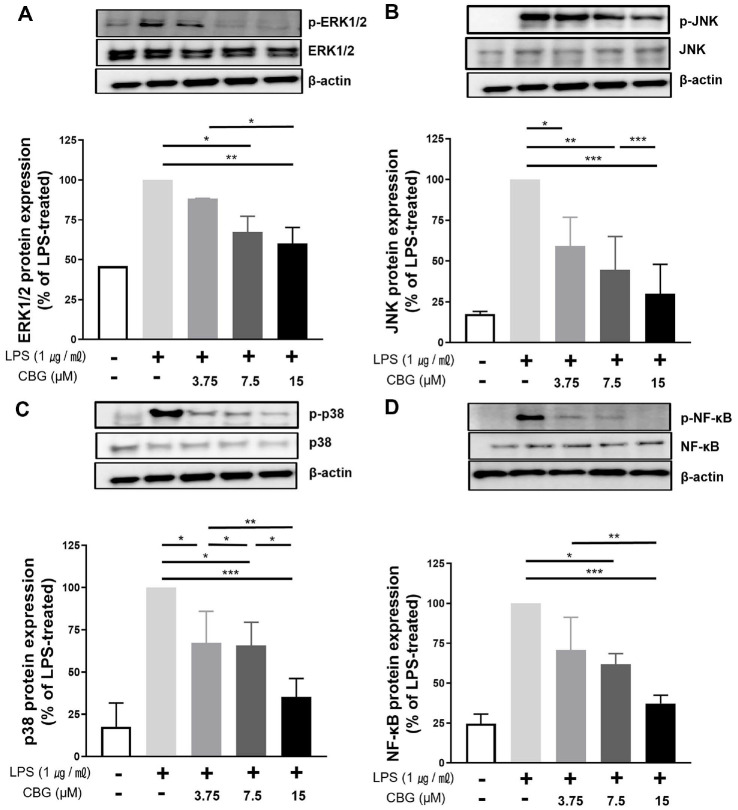
Inhibitory effect of CBG on phosphorylation of MAPK and NF-κB signaling pathways. All samples except the control were treated with 1 μg/ml of LPS. Western blot detection bands of each MAPK protein in groups treated with various concentrations of CBG compared with those of (**A**) p-ERK1/2, (**B**) p-JNK, and (**C**) p-p38 in the LPS-only treatment group. (**D**) Western blot detection bands of NF-κB protein in groups treated with various concentrations of CBG compared with those of p-NF-κB protein in the LPS-only treatment group. The *p*-values were calculated based on LPS-only data using ANOVA and Tukey’s post-hoc test (*p < 0.05, **p < 0.01, ***p < 0.001).

To elucidate the molecular mechanism of the anti-inflammatory effect of CBG, we investigated its impact on the MAPK signaling pathway. In LPS-stimulated RAW 264.7 cells, ERK1/2 phosphorylation was significantly inhibited by CBG, with reductions of 45.98 ± 0.11%, 67.33 ± 6.98%, and 60.21 ± 7.10% at concentrations of 3.75, 7.5, and 15 μM, respectively (Fig. 4A). JNK phosphorylation was also inhibited by 59.22 ± 14.42%, 44.57 ± 16.75%, and 29.96 ± 15.55% at the same concentrations of CBG (Fig. 4B). p38 phosphorylation was reduced by 67.12 ± 15.42%, 65.67 ± 11.20%, and 35.28 ± 9.40% at 3.75, 7.5, and 15 μM CBG, respectively (Fig. 4C). These results indicate that CBG shows anti-inflammatory responses by reducing the phosphorylation of key MAPK pathway proteins, thereby disrupting signal transduction involved in pro-inflammatory gene expression.

### CBG Suppresses LPS-Induced NF-κB Phosphorylation

To assess the regulatory role of CBG, we analyzed its effect on NF-κB phosphorylation, a key process controlling cytokine and inducible enzyme expression. In the untreated control group, the detected level of NF-κB phosphorylation was low (24.61 ± 4.20%). Phosphorylation of NF-κB was reduced in the LPS-treated group by 70.65 ± 16.91%, 61.87 ± 5.39%, and 37.24 ± 4.28% at CBG concentrations of 3.75, 7.5, and 15 μM, respectively (Fig. 4D).

### CBG Inhibits Acute Inflammatory Response Induced by λ-Carrageenan in Mice

**Fig. 5:**
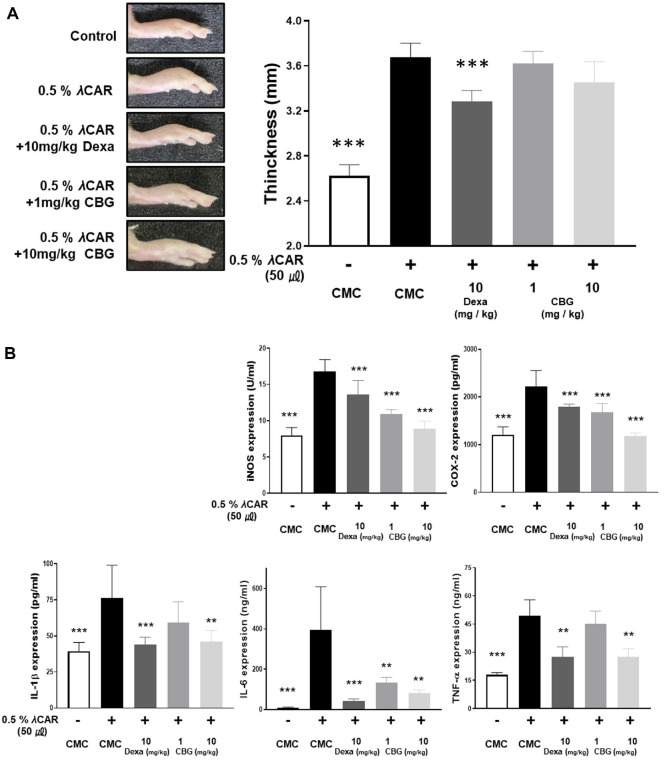
Effects of CBG on λ-carrageenan-induced edema in mice. Paw edema and inflammatory factor production in mice induced by λ-carrageenan. (**A**) Paw edema was measured using calipers 4 h after λ-carrageenan administration and compared with that in the control group. (**A**) Presents quantitative measurements and representative images of mouse paws to demonstrate the impact of orally administered CBG on λ-carrageenan–induced paw edema. (**B**) Inflammatory factor production was assessed using enzyme-linked immunosorbent assay (ELISA). P-values were calculated based on λ-carrageenan-only data using ANOVA and Tukey’s post-hoc test (*p < 0.05, **p < 0.01, ***p < 0.001).

The *in vivo* anti-inflammatory effect of CBG was confirmed through an acute inflammation model in mice. After inducing inflammation by administering 0.5% λ-carrageenan, oral administration of CBG was performed at dosages of 1 and 10 mg/kg. The thickness of the inflammation induced by 0.5% λ-carrageenan was not statistically significantly changed following oral administration of CBG (Fig. 5A). However, ELISA analysis of paw tissue protein extracts revealed that CBG significantly inhibited inflammatory marker expression (Fig. 5B). The level of iNOS, which increased to 16.84 ± 1.47 U/ml following λ-carrageenan injection, was reduced to 10.88 ± 0.59 and 8.89 ± 0.96 U/ml by oral administration of CBG at 1 mg/kg and 10 mg/kg, respectively. The concentration of COX-2 protein was 1207.23 ± 148.45 pg/ml (nontreated 0.5% λ-carrageenan). This level increased to 2222.36 ± 305.523 pg/ml after 0.5% λ-carrageenan treatment. Subsequent administration of 10 mg/kg dexamethasone, low-dose CBG, and high-dose CBG reduced the levels to 1787.94 ± 53.76, 1680.45 ± 170.72, and 1176.36 ± 62.12 pg/ml, respectively. Importantly, CBG administration showed a greater inhibitory effect on COX-2 and iNOS expression than the positive control, dexamethasone. When CBG was orally administered at doses of 1 and 10 mg/kg, IL-1β overexpression was inhibited from 76.43 ± 20.68 pg/ml to 59.17 ± 13.24, and 46.29 ± 6.83 pg/ml, respectively, in a dose-dependent manner. For IL-6, the level was reduced to 135.42 ± 22.75 ng/ml at 1 mg/kg of orally administered CBG, and further reduced to 83.46 ± 13.27 ng/ml at 10 mg/kg of orally administered CBG, which were comparable to the values observed in the λ-carrageenan-untreated group. TNF-α levels were not significantly reduced at 1 mg/kg CBG (44.99 ± 6.40 ng/ml), but there was a marked decrease at 10 mg/kg (27.48 ± 2.99 ng/ml). These results suggest that although CBG did not significantly suppress edema formation, it effectively reduced the protein of pro-inflammatory factors in paw edema mice with acute inflammation (Fig. 5).

## Discussion

Inflammation is a necessary function for life, but an uncontrolled inflammatory response causes serious diseases and destroys cells and tissues [23, 24]. Therefore, substances that suppress uncontrolled immune responses have pharmacological properties that can be utilized in various therapeutics.

LPS stimulates receptors such as CD14 and TLR4, and activates not only the MAPK pathway and NF-κB, but also various immune cytokines [24, 30]. CBG showed strong inhibition of NO activity (Fig. 1C) and inhibited the mRNA expression of inflammatory mediators induced by LPS (Fig. 2). Consistent inhibitory effects were observed at the protein level for iNOS, IL-1β, IL-6, and TNF-α; however, COX-2 protein expression did not show a reduction despite the inhibition at the mRNA level (Fig. 3). According to Lahtvee *et al*. [31], the correlation between mRNA and protein levels is limited; moreover, protein expression cannot be reliably inferred solely from mRNA levels. Furthermore, a decrease in mRNA levels does not necessarily lead to a corresponding decrease in protein levels owing to a phenomenon known as translational buffering [32]. Interestingly, this phenomenon appears to be specific to COX-2, as a similar pattern was also observed in our previous anti-inflammatory study using CBD in RAW 264.7 cells [33]. This observation suggests that cannabinoids may specifically modulate COX-2 expression, highlighting the need for further investigation.

NO is a key chemical marker of inflammation and inflammatory diseases, primarily produced by activated macrophages [34]. Exposure to bacterial components such as LPS upregulates iNOS, which in turn drives NO production [35]. During inflammation, NO production induces tumor inhibition, removal of invading pathogenic microorganisms, and regulation of the functional activity of immune cells; moreover, excessive NO production can adversely affect normal cellular functions [34]. Non-infectious inflammatory diseases exemplified by rheumatoid arthritis and osteoarthritis have been reported to trigger iNOS induction and nitric oxide generation [36]. Overproduction of NO results in the generation of peroxynitrite, a highly reactive nitrogen species that disrupts mitochondrial activity, injures cellular structures, and promotes neuronal loss [37]. These pathological events are implicated in neurodegenerative diseases, such as Alzheimer’s and Parkinson’s [37]. CBG may exert beneficial effects by significantly inhibiting the overproduction of NO, thereby offering therapeutic potential in diverse diseases associated with oxidative stress and inflammation [37].

ERK, JNK, and p38 form major branches of the MAPK pathway, which orchestrates critical biological processes including cell differentiation, proliferation, and migration [35]. Activation of MAPK modulates the expression of inflammatory cytokines, thereby contributing significantly to the inflammatory reaction [38]. Stimulation of macrophages by LPS induces phosphorylation of the IKK complex, leading to phosphorylation and ubiquitin-dependent degradation of IκBα, which in turn promotes the nuclear translocation of NF-κB p65 dimers [39]. Given that NF-κB regulates the transcription of various M1-associated inflammatory mediators, including TNF-α, IL-1β, IL-6, and COX-2, inhibition of this pathway represents a key molecular mechanism underlying the anti-inflammatory action of CBG observed in this study [39]. Given the central roles of the MAPK and NF-κB signaling cascades, their concurrent inhibition represents a promising therapeutic strategy for the development of anti-inflammatory agents. However, since the upstream IκBα degradation pathway of NF-κB phosphorylation was not directly examined in our experiments, further studies are warranted to elucidate the precise mechanisms by which CBG modulates this signaling cascade.

λ-Carrageenan is widely utilized to induce inflammation in animal models, particularly for evaluating novel anti-inflammatory and analgesic agents [40]. Injection of λ-carrageenan in rodents elicits a characteristic innate immune reaction, including edema, and nociceptive behavior [40]. In this study, an acute inflammatory response was induced by injecting λ-carrageenan into the right hind paw of the mice, and the anti-inflammatory effect of orally administered CBG was investigated (Fig. 5). Oral CBG treatment caused concentration-dependent suppression of inflammatory proteins isolated from paw tissues (Fig. 5B). However, CBG did not significantly reduce paw edema thickness in the λ-carrageenan model (Fig. 5A).

One possible explanation is a subtherapeutic dosage of orally administered CBG. Owing to its first-pass degradation, the bioavailability of CBG is significantly reduced, resulting in lower brain and tissue concentrations compared to systemic (*e.g.*, intravenous or intraperitoneal) administration [41]. Thus, a higher oral dosage may be required to achieve therapeutic effects. However, high-dose CBG has been reported to cause hepatotoxicity, necessitating caution in its dosage and application [42]. Additionally, the rapid metabolism of CBG following oral administration may result in a short duration of action, limiting its efficacy in tissues. Consistently, CBG was undetectable in plasma 24 h post-oral administration, suggesting limited pharmacokinetic stability [41]. These observations imply that CBG’s therapeutic potential may be attenuated when administered orally.

The therapeutic efficacy of major cannabinoid extracts is primarily mediated through the endocannabinoid system (ECS), a sophisticated regulatory system composed of receptors, enzymes, and endogenous ligands [5]. Key components of the ECS consist of cannabinoid receptors (CB_1_ and CB_2_), endocannabinoid ligands such as anandamide and 2-arachidonoylglycerol, transport proteins that mediate anandamide (AEA) trafficking, and enzymes responsible for both biosynthesis and catabolism [7]. Cannabinoids typically interact with two primary receptors (CB_1_ and CB_2_); however, CBG exhibits receptor selectivity, functioning as a partial activator of CB_2_ receptors while showing negligible direct activity at CB_1_ receptors [7]. Notably, CBG indirectly modulates CB_1_ receptor activity by inhibiting the reuptake of AEA, rather than engaging the receptor directly [7]. Selective activation of CB_2_ receptors by their ligands is associated with marked anti-inflammatory, antiviral, and immunomodulatory effects [44]. CB_2_ receptor activation regulates a broad array of immunoinflammatory signaling pathways, including those governing cytokines, chemokines, adhesion molecules, and prostanoids [44]. CB_2_ receptor antagonists also exhibit anti-inflammatory impacts by reducing neutrophil and macrophage infiltration and suppressing the expression of proinflammatory cytokines [7].

Beyond its action on cannabinoid receptors, CBG functions as a potent agonist of the TRPA1 channel, α_2_-adrenergic receptors, and 5-HT_1_A receptors [45-47]. TRPA1 is widely present in various immune-related non-neuronal cells, including macrophages, dendritic cells, T lymphocytes, and neutrophils, and has been linked to inflammatory and neuropathic conditions, highlighting its potential as a valuable pharmacological target [48–50]. Concurrently, activation of α_2_-AR suppresses proinflammatory cytokine production and promotes anti-inflammatory signaling cascades [51]. Taken together, these findings suggest that CBG may exert multifaceted anti-inflammatory effects through its combined activity on CBo receptors, TRPA1, and α_2_-AR, highlighting its potential as a pharmacological agent for immune and inflammatory disorders.

**Fig. 6:**
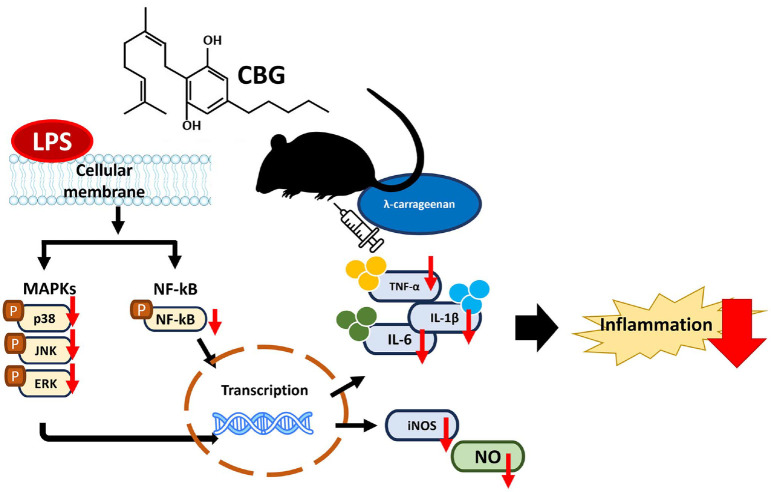
Proposed mechanism of inhibition of inflammatory signaling by cannabigerol in this study.

This study demonstrated that CBG, extracted and purified from the Korean cannabis cultivar ‘Pink Pepper,’ exerts significant anti-inflammatory effects by modulating key inflammatory mediators. CBG effectively suppressed the expression of pro-inflammatory cytokines, inflammatory mediators, and reduced activation of the MAPK signaling pathway, substantiating its capacity as a novel anti-inflammatory agent. A concise visual overview of the key findings of this study are depicted in Fig. 6.

Despite the clear anti-inflammatory activity demonstrated by CBG in both cellular and animal models, several limitations should be acknowledged in this study. Although the *in vivo* paw edema model provided useful insights into the acute anti-inflammatory effects of CBG, it reflects only a short-term inflammatory response. It is difficult to say whether CBG has potential as a treatment for diseases such as Parkinson's and rheumatism caused by chronic inflammation. Lastly, while this study focused on the MAPK and NF-κB signaling cascades, the involvement of other regulatory pathways cannot be excluded. Elucidating the interplay between these pathways and cannabinoid signaling will be important for a more comprehensive understanding of CBG’s anti-inflammatory mechanisms.

Caution is warranted in the oral administration of CBG owing to its limited bioavailability and the risk of hepatotoxicity at higher doses. Additionally, the psychoactive origin of cannabis may contribute to societal resistance and public concern; its association with addiction and abuse can lead to negative perceptions or misconceptions of its medical use. Nevertheless, CBG exhibits promising pharmacological characteristics as a broad-spectrum therapeutic agent, owing to its unique multi-receptor interactions. These findings underscore the therapeutic potential of CBG in inflammation-related diseases and advocate for further studies to elucidate its molecular mechanisms, optimize drug delivery systems, and expand its clinical applicability.

## Supplemental Materials

Supplementary data for this paper are available on-line only at http://jmb.or.kr.



## Figures and Tables

**Table 1:** Primers of inflammatory genes used in RT-PCR analysis.