# Supplementary material for: Cannabigerol Exerts In Vivo and In Vitro Anti-Inflammatory Effects via Inhibition of the MAPK and NF-κB Pathways
Source: J Microbiol Biotechnol. 2025 Dec 18;35:e2509034. doi: 10.4014/jmb.2509.09034 (PMC12740850; doi:10.4014/jmb.2509.09034)
Supplement: Supplementary file 1 [file jmb-35-e2509034-supple.pdf]

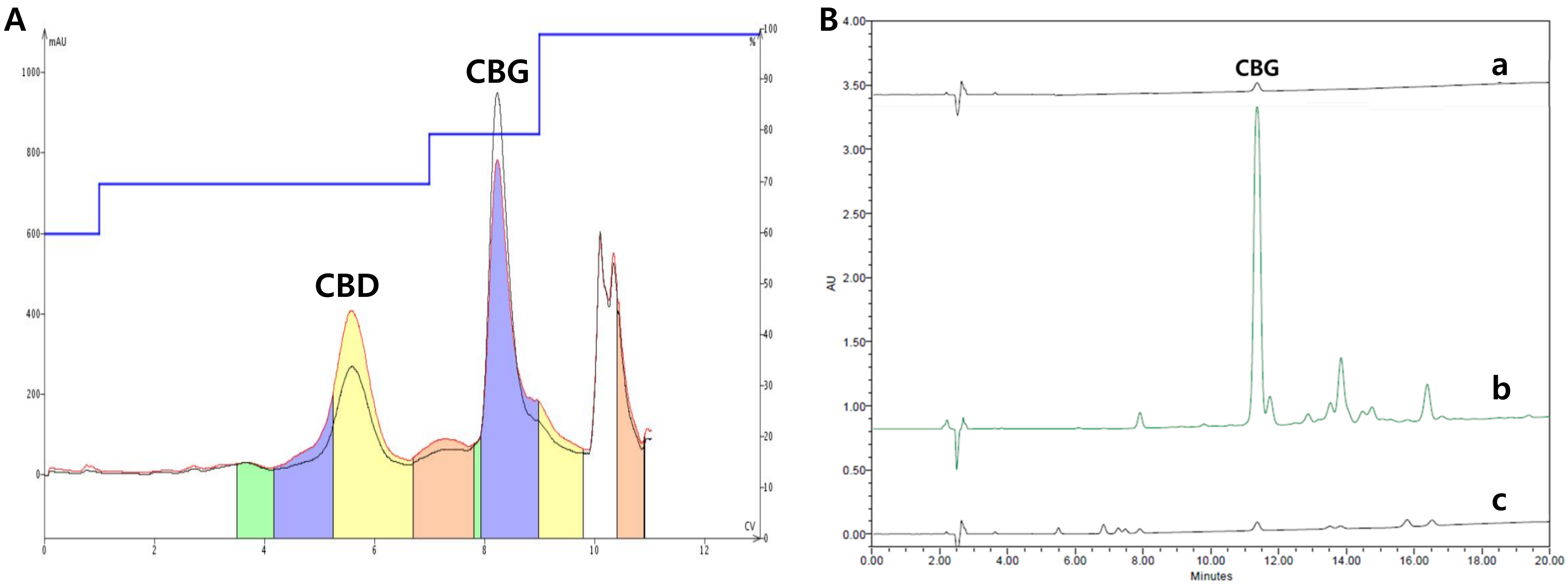

Supplementary Figure S1. MPLC and HPLC chromatograms of purified cannabigerol (CBG)
(A) MPLC chromatogram of cannabinoid fractions obtained from *Cannabis sativa L.* cv. *Pink Pepper*. Major peaks of CBD and CBG were detected at approximately 5.3 CV and 8.1 CV, respectively.
(B) HPLC chromatograms confirming CBG purity. a) crystallized CBG (≥99% purity); b) CBG oil after MPLC purification; c) analytical CBG standard.
